# In reply: efficacy of a new videolaryngoscope; what we should assess?

**DOI:** 10.1007/s00540-012-1535-y

**Published:** 2012-12-15

**Authors:** Takashi Asai, Tomoyuki Saito, Yasuhisa Okuda

**Affiliations:** 1 Department of Anesthesiology, Takii Hospital, Kansai Medical University, 10-15 Fumizono-cho, Moriguchi, Osaka 570-8507 Japan; 2 Department of Anesthesiology, Koshigaya Hospital, Dokkyo Medical University, 2-1-50 Minamikoshigaya, Koshigaya, Saitama 343-8555 Japan

To the Editor:

We thank Dr. Meng and colleagues for their constructive comments to our study [1]. Videolaryngoscopes have been shown to be useful in patients with and without difficult airways [2–5], but their efficacies may differ [2]. To assess the usefulness of each new device, we need to address three major factors: the device (efficacy), the patient (degree of difficult airway), and the performer (expertise). In addition to these major factors, as Meng and colleagues point out, we may need to assess the efficacy of supportive measures (such as pressure on the neck or use of a stylet). It would be ideal to assess all of these, but it is often impractical to standardize all factors (other than the device factor). We considered that because it would be difficult and may be unethical to standardize the patient factor in real patients, we chose to standardize the patient factor using a manikin with four different simulated difficult airways.

Regarding the expertise of the performer, we stated the reasoning for choosing residents and their equal proficiency in our article [1]:

It is known that the expertise of the anesthesiologist will affect the success rate of tracheal intubation. We considered that, for experienced anesthesiologists, they would be able to intubate the trachea using either a Macintosh laryngoscope or a fibreoptic bronchoscope, whereas for less experienced anesthesiologists (but with minimum skills), videolaryngoscopes may be regarded as the first choice when tracheal intubation using a Macintosh laryngoscope has failed. The participants had minimum skills with the Macintosh laryngoscopes, because in a manikin with normal airway, there were no significant differences in the success rate of tracheal intubation between the VLP-100, the AWS, and the Macintosh [1].

Meng and colleagues state that the difference in laryngoscopic views among three laryngoscopes was stated as a final endpoint of performance comparison, and comparing the views obtained with direct and video laryngoscopes is not an entirely appropriate comparison. We agree with their comments on the uncertainty regarding the use of the Cormack–Lehane score for assessing the efficacy of videolaryngoscopes, and this is why we did NOT use the view of the glottis as the primary endpoint. We clearly state that the main aim of our study was to compare success rates of tracheal intubation between the three laryngoscopes, and tracheal intubation was the primary endpoint [1].

As Meng and colleague imply, there are many factors that we did not study: for example, there is still insufficient evidence to judge whether tracheal intubation using a videolaryngoscope is less likely to traumatize the airway or to prolong apnea time [2]. We need to continue carrying out randomized controlled trials and reporting large case series, together with some tips for effective use, to establish the true role of each videolaryngoscope in patients with and without difficult airways.
